# Gastric metaplasia of posterior urethral polyp: a case report

**DOI:** 10.1186/1757-1626-2-9119

**Published:** 2009-11-30

**Authors:** Mehdi Jaidane, Adnen Hidoussi, Adel Slama, Wissem Hmida, Nabil Ben Sorba, Faouzi Mosbah

**Affiliations:** 1 Urology Department, Sahloul University Hospital, Sousse, Tunisia

## Abstract

Congenital polyps of the posterior urethra are rare lesions, and have often been described in boys. The polyps are benign lesion usually lined by a transitional epithelium, but cases of squamous or intestinal metaplasia have been reported.

We report a case of a 10 years old boy referred for hematuria and dysuria.

The voiding cysto urethrogram revealed a filling defect in the posterior urethra. At cystourethroscopy, a polyp of the posterior urethra was found and resected transurethrally. Histological examination showed a polyp with a fibro muscular core covered by focal gastric metaplasia with fundic gland. To our knowledge this is the first reported case of gastric metaplasia of urethral polyp.

## Introduction

Urethral polyps are a rare cause of bladder outlet obstruction in the pediatric age group. They are benign fibroepithelial polyps usually diagnosed in the first decade. They have been described often in boys and the posterior urethra is the predominant location [1].

Most patients present with varying degrees of irritative and obstructive voiding symptoms and hematuria [2].

## Case presentation

A 10 year old boy was referred to our hospital with complaints of dysuria and hematuria. He had a 6-month history of progressive loss of force and interruption of his urinary stream. He had also a recent history of urinary tract infection treated by antibiotics. Clinical examination and ultrasonography revealed no abnormalities.

A voiding cysto urethrogram showed no reflux or urethral valves but revealed a filling defect in the posterior urethra. Cystourethroscopy revealed the presence of 0,5 cm polyp arising from the posterior urethra. The polyp was resected in its entirety at the base using a 9F resectoscope.

**Figure 1:**
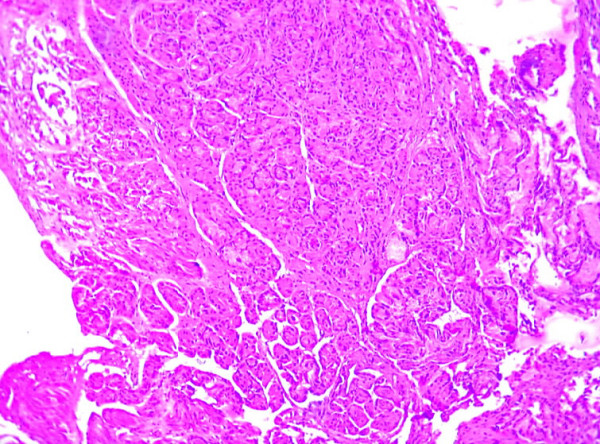
**Histopathology (original magnification ×100) showing polyp with a fibro muscular core covered by gastric metaplasia with heterotopic fundic glands**.

**Figure 2:**
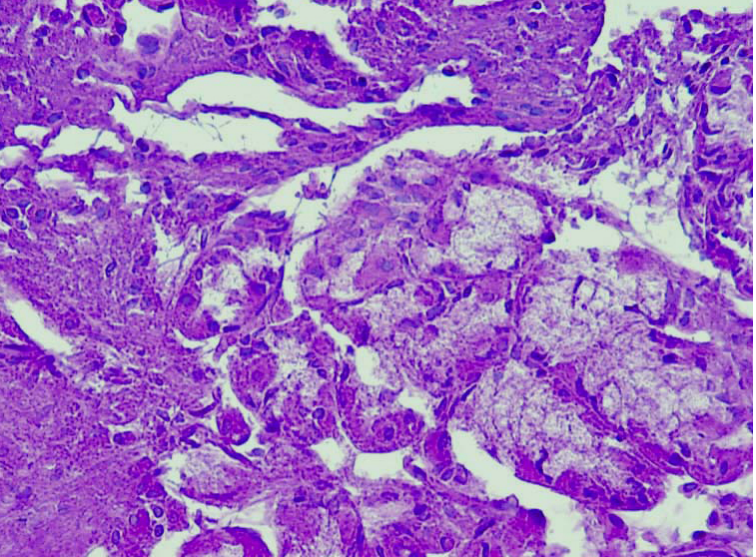
**Histopathology (original magnification ×400) showing fundic glands**.

Histopathological examination showed the lesion to be a benign fibroepithelial polyp covered by transitional epithelium with focal areas of gastric metaplasia with fundic glands (figure 1 and 2). Under the epithelium, there was edematous lamina propria with smooth muscle cells. The patient was discharged home two days after surgery, with an uneventful postoperative course. 4 years after surgery, the boy had no urinary symptom and no recurrence.

## Discussion

Polyps derived from the lower urinary tract are not as frequent when compared with those derived from the upper urinary tract [3]. Polyps of the posterior urethra are rare benign fibro epithelial growths, usually covered by transitional epithelium but cases of squamous or intestinal metaplasia have been reported [4,5].

The presence of large polyps in healthy newborns and infants is a strong argument in favor of a congenital origin. It has been proposed that they may arise from mesonephric remnants [6].

Concerning metaplasia of the urothelium, three basic types are described: squamous, intestinal, and nephrogenic metaplasia [7].

Intestinal metaplasia of the superficial epithelium of the prostatic urethra was reported, and can be associated with dysplasia [8].

Gastric metaplasia is extremely rare and has been previously recognized in the urothelium of the ureter [9] and the bladder [10], but never in the urethra.

To our knowledge, the present case is the first reported case of urethral gastric heterotopia.

The etiology of the metaplasia of the urothelium is still controversial. Congenital, irritative, infectious, obstructive and traumatic causes have been proposed [4]. Metaplasia is thought to be reactive process to urothelial injury induced by chronic irritation. Maternal estrogen during pregnancy was also thought to be involved [11].

In our observation, gastric metaplasia of the posterior urethral polyp can be linked to obstruction and chronic inflammation.

Transurethral resection has provided an adequate therapeutic approach in this case. Considerable caution must be taken because of the proximity of the external sphincter to the verumontanum.

Other surgical options include Bugbee fulguration of the polyp, laser excision and open suprapubic transvesical excision [6].

Regardless of the treatment, there have been no reported recurrences when the polyp has been removed completely [12].

## Consent

Written informed consent was obtained from the parents of the patient for publication of this case report and accompanying images. A copy of the written consent is available for review by the Editor-in-Chief of this journal.

## Competing interests

The authors declare that they have no competing interests.

## Authors' contributions

MJ and AH collected the data and literature review, and wrote the manuscript. AS, WH and NBS revised and provided comments on the manuscript. FM was the attending doctor, carried out the surgical procedure and literature review. All authors read and approved the final manuscript.
